# Review of Remote Consultations in Learning Disability During COVID Pandemic

**DOI:** 10.1192/bjo.2022.415

**Published:** 2022-06-20

**Authors:** Charvi Saraswat, Catherine Bright

**Affiliations:** Aneurin Bevan University Health Board, Newport, United Kingdom

## Abstract

**Aims:**

The aim of this project was to assess the efficacy of remote consultations in patients with Learning Disability (LD). In Aneurin Bevan University Health Board, teleconsultation or “Attend Anywhere” (Video) platforms are the two types of remote consultation that is being offered.

**Methods:**

A 9- point Questionnaire was used to assess the efficiency of the consultation. During consultation (Either telephone or attend anywhere), data were collected by the consultants by answering the questionnaire. 23 clinics organised between 04/06/2020 to 23/06/2020 for Service Users' (SU) follow-up.

The following key points were covered in the questionnaire:

1. Mode of consultation- telephone or attend anywhere
2. Presence of the SU
3. Introduction
4. Availability of information (patient notes/shared drives) prior to consultation
5. Time constraints
6. Information not covered due to lack of face-to-face consult
7. Technical difficulties
8. Expectations from SU
9. Feedback from SU

**Results:**

1. The most common mode of consultation was via telephone (70%), followed by Attend Anywhere (30%)
2. The majority of conversations were with SU's family or carers (70%); consultations with SU were only 30%. SU were unable to attend the consultation due to: Communication difficulty (26%), follow-ups provided by carer's/family's feedback (21.7%), SU away due to physical health reasons, or in day care (17.3%).
3. Introductions were done and sufficient information regarding the service users were available in all consultations.
4. Expectation of SU/carers/family was with regards to medication review (43%).
5. 52% of remote consultation were disrupted due to technical problems, for instance call drops and line disruptions, microphone issues and SU not being able to use attend anywhere because of its complexity.

**Conclusion:**

It was demonstrated that remote consultation could possibly be most effective for medication reviews or regular follow-up appointments.

Some of the aspects that were not covered due to the shortcomings of remote consultations were:

- Difficulty in assessing body language and facial expressions
- Difficulty in assessing the level of function of SU
- Unable to monitor physical health parameters
- Difficulty in picking up non-verbal cues, and assess eye contact to ascertain mood component of presentation.

In summary it seems in the early stages of the pandemic, telephone consultation was the predominant form of remote consultation. Further work would be useful to obtain the views of people with LD, their carers and families as to which form of consultation would be their preference and whether remote consultation is acceptable for this patient group.

